# Predictors of functional outcome at 3 months in ischemic stroke patients with discharge disability following endovascular therapy: a multi-center observational cohort study of 836 patients

**DOI:** 10.1007/s00701-025-06758-3

**Published:** 2026-01-06

**Authors:** Mohammad Mofatteh, Xiao Xiao, Yimin Chen, Junyi Hu, Mingzhu Feng, Jicai Ma, Lue Chen, Sijie Zhou, Xiuling Zhang, Zunbao Xu, Jiale Wu, Yongting Zhou, Yuzheng Lai, Wenhong Peng

**Affiliations:** 1 School of Medicine, Dentistry and Biomedical Sciences, Queen’s University Belfast, Belfast, UK; 2 Department of Neurosurgery, Neuro International Collaboration (NIC), London, UK; 3 Department of Medicine, National University Hospital, Singapore, Singapore; 4 Department of Medicine, Yong Loo Lin School of Medicine, National University of Singapore, Singapore, Singapore; 5 Division of Neurology, Department of Medicine, National University Hospital, Singapore, Singapore; 6 Department of Statistics and Data Science, Faculty of Science, National University of Singapore, Singapore, Singapore; 7 Department of Neurology and Advanced National Stroke Center, Foshan Sanshui District People’s Hospital (Sanshui Hospital, Zhujiang Hospital, Southern Medical University), Foshan, Guangdong Province China; 8 Department of Neurology, The Affiliated Yuebei People’s Hospital of Shantou University Medical College, Shaoguan, Guangdong Province China; 9 Department of Neurology, The Eighth Affiliated Hospital of Southern Medical University (The First People’s Hospital of Shunde, Foshan), Foshan, Guangdong Province China; 10 Department of Surgery of Cerebrovascular Diseases, First People’s Hospital of Foshan, Foshan, Guangdong Province China; 11 Neuromedical Center Ward 2, The Affiliated Panyu Central Hospital, Guangzhou Medical University, Guangzhou, China; 12 Medical Intern, Department of Neurology, Foshan Sanshui District People’s Hospital (Sanshui Hospital, Zhujiang Hospital, Southern Medical University), Foshan, China; 13 Department of Neurology, Huadu District People’s Hospital of Guangzhou, Guangzhou, China; 14 Medical Intern, Foshan Sanshui District People’s Hospital (Sanshui Hospital, Zhujiang Hospital, Southern Medical University), Foshan, China; 15 Department of Neurology, Guangdong Provincial Hospital of Integrated Traditional Chinese and Western Medicine (Nanhai District Hospital of Traditional Chinese Medicine of Foshan City), Foshan, Guangdong Province China; 16 Department of Neurology and Advanced National Stroke, Affiliated hospital of Shaoguan University, Shaoguan, Guangdong Province China

**Keywords:** Endovascular thrombectomy, Functional outcome, Stroke recovery, Prognosis, Reperfusion

## Abstract

**Background:**

Endovascular therapy (EVT) is the standard of care for acute ischemic stroke due to large vessel occlusion. While predictors of 90-day functional outcome are well-established, the determinants of functional recovery remain less clearly defined in the post-discharge period for patients with initial disability. We aimed to identify the predictors of functional outcome at 3 months in patients who underwent EVT and were discharged with an unmet need for recovery (modified Rankin scale (mRS) score > 2).

**Methods:**

A multi-center, observational cohort study was conducted using data from the Big Data Observatory Platform for Stroke in China. We included 836 patients from eight comprehensive stroke centers (August 2018 – December 2024) who received EVT, had a pre-stroke mRS of 0–2, and had an mRS > 2 at discharge. The primary outcome was functional outcome at 3 months post-EVT, defined as an mRS score of 0–2. Univariate and multivariate logistic regression analyses were performed to identify independent predictors.

**Results:**

Of the 836 patients, 151 (18.1%) achieved a favorable functional outcome (mRS 0–2) at 3 months. In univariate analysis, the favorable outcome group was significantly younger, had a lower pre-EVT NIHSS, a lower rate of atrial fibrillation, a higher rate of intravenous thrombolysis, a higher rate of complete recanalization (mTICI 3), and a lower rate of parenchymal hematoma (PH) (all *p* < 0.05). Multivariate regression confirmed four independent predictors: younger age (aOR: 0.973; 95% CI: 0.958–0.989; *p* = 0.001), lower pre-EVT NIHSS (aOR: 0.940; 95% CI: 0.912–0.968; *p* < 0.001), complete recanalization (aOR: 1.921; 95% CI: 1.305–2.826; *p* = 0.001), and absence of PH (aOR: 0.424; 95% CI: 0.235–0.768; *p* = 0.005).

**Conclusion:**

A significant proportion of patients discharged with disability experiences meaningful functional recovery by 3 months post-EVT. The key predictors of this subsequent recovery are younger age, milder initial stroke severity, complete reperfusion, and the avoidance of hemorrhagic complications.

## Introduction

Ischemic stroke remains a leading cause of long-term adult disability and mortality worldwide [28, 36]. The advent of endovascular therapy (EVT), particularly mechanical thrombectomy, has revolutionized the management of acute ischemic stroke (AIS) secondary to large vessel occlusion (LVO), offering unprecedented rates of vascular recanalization and improved clinical outcomes [11, 38, 40]. Landmark randomized controlled trials have unequivocally established EVT, in conjunction with best medical management, as the standard of care for eligible patients, demonstrating significant benefits in functional independence compared to medical therapy alone [5, 13, 14].

The primary efficacy measure in these pivotal studies, and in contemporary clinical practice, is the degree of functional recovery at 90 days, typically assessed using the modified Rankin Scale (mRS) [18, 32, 35, 46]. A favorable outcome is conventionally defined as a mRS score of 0–2, denoting functional independence [31, 49]. Consequently, a substantial body of research has been dedicated to identifying predictors of this short-to-medium term functional status, with well-established factors including younger age, lower baseline stroke severity as measured by the National Institutes of Health Stroke Scale (NIHSS), successful reperfusion (modified Thrombolysis in Cerebral Infarction, mTICI 2b-3), and the absence of significant hemorrhagic complications [10, 19, 22, 26, 27, 51, 52, 54].

However, a significant proportion of patients are discharged from acute care with persistent functional dependence (mRS > 2). The clinical trajectory of these patients is dynamic and particularly consequential, yet it remains less well understood. While discharge planning and early post-acute care are often informed by the 90-day prognosis, there is a critical gap in understanding the determinants of functional outcomes specifically between discharge and the 3-month mark for this dependent population [12, 16, 17, 21, 24, 29, 30, 41]. This post-discharge phase is critical as it reflects the period of most intensive rehabilitation and the patient's initial reintegration into their community, directly impacting long-term quality of life [4]. The factors that govern recovery during this subsequent period may differ from those influencing the initial 90-day window, being potentially more influenced by the neurological reserve established during the acute phase, as well as rehabilitation access, social support structures, and long-term complication management [2, 16, 45].

Despite its clinical importance for prognostication and rehabilitation planning, the predictors of continued recovery trajectory specifically after discharge in functionally dependent EVT patients remain less comprehensively characterized. Most studies conclude their follow-up at 90 days, often without focusing on the subpopulation that was dependent at discharge, leaving a gap in knowledge regarding which of these patients are most likely to experience further improvement [33, 39, 43]. Understanding these predictors is essential for optimizing long-term care pathways, setting realistic patient expectations, and efficiently allocating rehabilitation resources.

Therefore, to address this gap in the literature, we designed a multi-center observational study. Our primary objective was to identify the key baseline, clinical, and procedural predictors associated with achieving functional independence by 3 months specifically in patients who were discharged with functional dependence (mRS > 2) after undergoing EVT for acute ischemic stroke. By elucidating these factors, our findings aim to contribute to more stratified and personalized long-term management strategies for this important subgroup of stroke survivors.

## Methods

### Study design and population

This was a multi-center, observational, retrospective cohort study conducted using data from the Big Data Observatory Platform for Stroke in China. We included consecutive patients with AIS secondary to LVO who underwent EVT at one of eight comprehensive stroke centers in China between August 2018 and December 2024.

The study inclusion criteria were as follows: 1) age ≥ 18 years; 2) pre-stroke functional independence, defined by a mRS score of 0–2; 3) presentation within 24 h of symptom onset (or from the last known well time for patients with unwitnessed onset), 4) pre-treatment Alberta Stroke Program Early Computed Tomography Score (ASPECTS) of ≥ 6 on non-contrast CT imaging; 5) underwent EVT (mechanical thrombectomy with or without prior intravenous thrombolysis). The exclusion criteria were applied as follows: 1) pre-EVT neurological deficit deemed too mild (NIHSS score ≤ 6); 2) in-hospital mortality; 3) discharge to a rehabilitation or chronic care facility with an already-favorable functional status (mRS score 0–2 at discharge), as the study focus was on predicting recovery in those with unmet need; 4) lost to 3-month follow-up.

The time of discharge was determined by the treating clinical team based on standard clinical criteria, including medical stability and completion of acute stroke workup. The median time from EVT to discharge was 14.0 days.

### Data collection and variables

Relevant data were obtained retrospectively from the prospectively maintained databases of each participating hospital's stroke registry, which were integrated into the central Big Data Observatory Platform. The collected variables included baseline demographics (age and sex), vascular risk factors (hypertension, diabetes mellitus, dyslipidemia, coronary artery disease, atrial fibrillation, previous stroke, and smoking status), stroke severity and imaging (pre-EVT NIHSS score and pre-EVT ASPECTS), treatment parameters (administration of intravenous thrombolysis (IVT) and onset-to-puncture time (OPT)), and procedural outcome (degree of reperfusion post-thrombectomy, assessed using the mTICI scale). Successful reperfusion was defined as mTICI 2b–3, and complete reperfusion was defined as mTICI 3. Safety outcome was the occurrence of parenchymal hematoma (PH), as defined by the European Cooperative Acute Stroke Study (ECASS) criteria [25].

Intravenous thrombolysis (IVT), when administered, was primarily performed with Alteplase at a standard dose of 0.9 mg/kg (maximum 90 mg), with 10% as an initial bolus, followed by a 60-min infusion of the remaining 90%, in accordance with national guidelines. IVT was administered prior to EVT (bridging therapy) in all eligible patients presenting within the approved time window.

### Outcome measures

The primary outcome of the study was the functional outcome, defined as the functional status at 3 months post-EVT. Outcome was assessed during routine clinical follow-up visits or via standardized telephone interviews by certified neurologists or research nurses blinded to the study hypothesis. Functional outcome was measured using the mRS. A favorable functional outcome was defined as an mRS score of 0–2 (functional independence). An unfavorable outcome was defined as an mRS score of 3–6 (functional dependence or death).

### Statistical analysis

Statistical analyses were performed using IBM SPSS Statistics version 27 (IBM Corp., Armonk, NY, USA). Continuous variables were tested for normality using the Shapiro–Wilk test. Normally distributed data are presented as mean ± standard deviation (SD) and were compared using the independent Student’s t-test. Non-normally distributed data are presented as median with interquartile range (IQR) and were compared using the Mann–Whitney U test. Categorical variables are presented as counts and percentages and were compared using the Chi-square or Fisher’s exact test, as appropriate. To identify independent predictors of a favorable functional outcome (mRS 0–2 at 3 months), variables with a *p*-value < 0.1 in the univariate analysis (as indicated in Table 1) were entered into a multivariate binary logistic regression model using the backwards stepwise (likelihood ratio) method. This relaxed threshold was used to minimize the risk of excluding potential confounding variables from the multivariate model. The results of the regression analysis are presented as adjusted odds ratios (OR) with their corresponding 95% confidence intervals (CI). A two-tailed *p*-value of < 0.05 was considered statistically significant for all final analyses.

**Table 1:** Baseline characteristics of patients

	mRS 3–6 at 90 days (*n*, %)	mRS 2 at 90 days (*n*, %)	*P*
Number	685	151	
Age (y), (median, IQR)	69.0 (59.0–76.0)	65.0 (54.0–74.0)	0.001**
Male sex	422 (61.61)	101 (66.89)	0.225
Hypertension	463 (67.59)	97 (64.24)	0.428
Diabetes mellitus	186 (27.15)	35 (23.18)	0.316
Coronary artery diseases	122 (17.81)	23 (15.23)	0.449
Smoker	167 (24.38)	44 (29.14)	0.223
Atrial fibrillation	263 (38.39)	45 (29.80)	0.048*
Previous stroke	152 (22.19)	25 (16.56)	0.125
Dyslipidemia	143 (20.88)	42 (27.81)	0.063
Pre-EVT NIHSS (median, IQR)	18.000 (14.0- 22.0)	15.000 (11.0–19.0)	0.000**
Pre-EVT ASPECTS (median, IQR)	8.000 (7.0- 10.0)	9.000 (8.0- 9.0)	0.355
Intravenous thrombolysis	232 (33.87)	64 (42.38)	0.048*
OPT (median, IQR), min	320.00 (207.5–525.5)	330.000 (210.0- 570.0)	0.685
Complete recanalization	379 (55.33)	103 (68.21)	0.004**
Parenchymal hematoma	131 (19.21)	14 (9.27)	0.004**

*ASPECTS* The Alberta Stroke Program Early CT Score, *EVT* endovascular thrombectomy, *IQR* interquartile range, *NIHSS* National Institutes of Health Stroke Scale, *OPT* onset-to-puncture time. *Denotes significance

## Results

### Patient selection and baseline characteristics

**Fig. 1:**
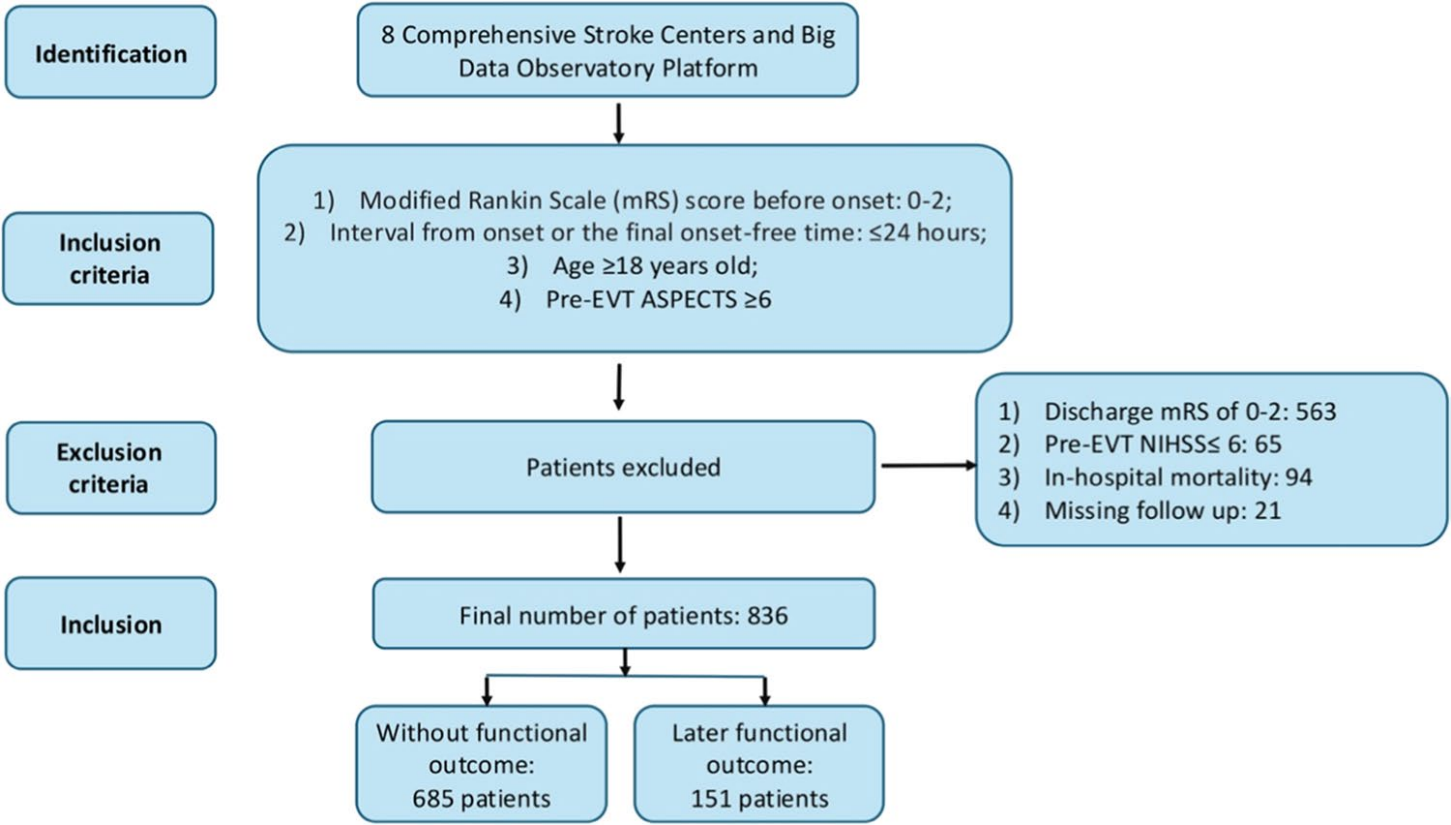
A flowchart demonstrating patient selection in the current study

From August 2018 to December 2024, a total of 1579 patients who underwent EVT for acute ischemic stroke were initially screened across the eight participating centers. After applying the exclusion criteria, 836 patients were included in the final analysis. Exclusions were for the following reasons: 65 patients had a pre-EVT NIHSS ≤ 6, 94 died during the hospital stay, 563 had a favorable functional status (mRS 0–2) at discharge, and 21 were lost to the 3-month follow-up (Fig. 1).

The cohort was stratified based on the 3-month functional outcome. A total of 151 patients (18.1%) achieved a favorable functional outcome (mRS 0–2), while 685 patients (81.9%) had an unfavorable outcome (mRS 3–6).

The baseline characteristics of the two groups are summarized in Table 1.

Compared to the unfavourable outcome group, patients with a favorable outcome were significantly younger (median 65.0 vs. 69.0 years, *p* ≤ 0.001) and had a lower median pre-EVT NIHSS score (15.0 vs. 18.0, *p* ≤ 0.001). The prevalence of atrial fibrillation was significantly lower in the favorable outcome group (29.80% vs. 38.39%, *p* = 0.048). A significantly higher proportion of patients in the favorable outcome group received intravenous thrombolysis (42.38% vs. 33.87%, *p* = 0.048) and achieved complete recanalization (mTICI 3) (68.21% vs. 55.33%, *p* = 0.004). Furthermore, the occurrence of parenchymal hematoma was significantly less frequent in the favorable outcome group (9.27% vs. 19.21%, *p* = 0.004). No other significant differences were observed in other vascular risk factors, sex, pre-EVT ASPECTS, or onset-to-puncture time between the two groups.

Regarding mortality, the overall rate of death (mRS 6) at 3 months was 26.91%. As expected, all deaths occurred in the unfavorable outcome group, constituting 32.85% of those patients. No patients in the favorable outcome group (mRS 0–2) had died by the 3-month follow-up.”

### Multivariate analysis for predictors of functional outcome

Variables with a *p*-value < 0.1 in the univariate analysis (age, atrial fibrillation, pre-EVT NIHSS, intravenous thrombolysis, complete recanalization, and parenchymal hematoma) were included in a multivariate binary logistic regression model to identify independent predictors of a favorable functional outcome at 3 months.

As presented in Table 2, the analysis identified four independent predictors:

1. Younger age (adjusted OR: 0.973 per year increase; 95% CI: 0.958–0.989; *p* = 0.001)
2. Lower pre-EVT NIHSS Score (adjusted OR: 0.940 per point increase; 95% CI: 0.912–0.968; *p* ≤ 0.001)
3. Complete recanalization (mTICI 3) (adjusted OR: 1.921; 95% CI: 1.305–2.826; *p* = 0.001)
4. Absence of parenchymal hematoma (adjusted OR: 0.424; 95% CI: 0.235–0.768; *p* = 0.005)

**Table 2:** Factors associated with functional outcome at 3 months after multivariate regression

Variables	Coefficient	*p*	OR	OR of 95% CI
Age	−0.027	0.001*	0.973	0.958—0.989
Atrial fibrillation	−0.065	0.763	0.937	0.615—1.428
Pre-EVT NIHSS	−0.062	0.000*	0.940	0.912—0.968
IVT Thrombosis	0.319	0.095	1.375	0.946—1.999
Complete Recanalization (mTICI = 3)	0.653	0.001*	1.921	1.305—2.826
Parenchymal hematoma	−0.857	0.005*	0.424	0.235—0.768

*EVT* endovascular thrombectomy, *IQR* interquartile range, *mTICI* modified treatment in cerebral infarction, *NIHSS* National Institutes of Health Stroke Scale. *Denotes significance

Atrial fibrillation and intravenous thrombolysis were not independent predictors in the multivariate model (AF: *p* = 0.763; IVT: *p* = 0.095).

The modal outcome was an mRS score of 4, indicating moderately severe disability.

## Discussion

This multi-center, observational study provides valuable insights into the determinants of functional recovery in the post-discharge period for ischemic stroke patients who were functionally dependent at discharge. Our key finding is that a significant proportion of these patients (18.1%) who were dependent at discharge (mRS > 2) achieved functional independence (mRS 0–2) by the 3-month mark. This highlights that the recovery trajectory does not end at discharge for a clinically relevant minority. We identified four independent predictors of this meaningful recovery: younger age, lower baseline NIHSS, complete recanalization (mTICI 3), and the absence of parenchymal hematoma.

The identification of these predictors specifically within a cohort of patients who all shared a common starting point of disability at discharge is a key strength of our study. It moves beyond predicting the initial 90-day outcome and instead helps answer the clinically pressing question: "Among my patients leaving the hospital disabled, who has the greatest potential for further meaningful recovery?".

The finding that younger age is a powerful predictor of better long-term outcome is one of the most consistent themes in stroke literature, which is strongly reinforced by our results [37]. This neuroplastic potential appears to be a crucial driver for recovery even after the acute phase, enabling a greater response to rehabilitation in the months following discharge [1, 44]. Similarly, a lower pre-intervention NIHSS score, representing a smaller initial ischemic insult, logically portends a higher potential for recovery [20, 42]. Our results confirm that a smaller initial ischemic insult not only predicts a better outcome at 90 days but also identifies a greater latent potential for improvement specifically among those who have not yet recovered by the time of discharge.

The strong association between complete recanalization (mTICI 3) and favorable outcome underscores the paramount importance of achieving the highest possible degree of reperfusion. While successful reperfusion (mTICI 2b-3) is an established goal, our analysis adds to a growing body of evidence suggesting that there is a significant clinical gradient between "successful" (mTICI 2b) and "complete" (mTICI 3) reperfusion [15, 34, 53]. A TICI 3 result ensures maximal salvage of the ischemic penumbra, minimizes the final infarct volume, and provides a more substantial neuroanatomical substrate upon which post-stroke rehabilitation can act [6]. Our study suggests that the benefit of complete reperfusion extends beyond preventing early deterioration; it actively enables continued neurological improvement well after discharge by maximizing the preserved neuroanatomical substrate upon which post-stroke rehabilitation can act.

Conversely, the development of parenchymal hematoma was a potent negative predictor, associated with a 58% reduction in the odds of achieving a good later outcome. This complication appears to create a profound and lasting barrier to recovery, significantly diminishing the potential for the functional gains that typically occur in the post-acute period [7, 23]. Parenchymal hematoma typically signifies severe blood–brain barrier disruption and irreversible tissue injury, often leading to clinical deterioration that nullifies the benefits of recanalization and creating a new, often prohibitive, barrier to functional recovery [3, 9].

Interestingly, while intravenous thrombolysis (IVT) showed a positive trend in univariate analysis, it was not an independent predictor in the multivariate model. This suggests that its benefit may be largely mediated through its ability to facilitate higher rates of complete recanalization, an effect that has been observed in other studies [8, 47]. Furthermore, the loss of statistical significance for atrial fibrillation (AF) in multivariate analysis indicates that its negative impact on outcome is likely conveyed through other mechanisms, such as an association with more severe baseline strokes, larger clot burdens, or a higher comorbidity profile, which was accounted for by other variables in the model [48, 50].

## Limitations

Our study has several limitations. Firstly, its observational and retrospective design inherently carries risks of unmeasured confounding despite our multivariate adjustments. Factors not captured in our registry, such as the intensity, quality, and type of post-discharge rehabilitation, social support networks, socioeconomic status, and specific post-discharge complication management, likely play a crucial role in long-term recovery. [32] Secondly, while multi-center, the data originated from a single healthcare system in China, which may limit the generalizability of our findings to other populations with different stroke care pathways. Finally, the 3-month follow-up, while standard, may not capture the full extent of long-term recovery, which can continue for up to a year in some patients. Third, the mTICI score was categorized as 0, 1, 2a, 2b, and 3. The potentially distinct category of mTICI 2c was not consistently recorded across all participating centers, precluding a separate analysis of its effect compared to mTICI 2b and 3.

## Conclusion

In conclusion, our study demonstrates that for patients discharged with disability after EVT, functional recovery is a dynamic process that continues meaningfully for a substantial minority. We have identified key factors that predict this subsequent improvement to functional independence. These findings reinforce that functional status at discharge should not be viewed as a definitive endpoint for prognostication. Instead, in patients discharged with an mRS > 2, factors such as younger age, milder initial stroke severity, complete reperfusion, and the absence of hemorrhagic complication can help clinicians identify those with a significant potential for further recovery by 3 months, thereby guiding rehabilitation strategies and patient counseling.

## Data Availability

Relevant data and materials are available by contacting the corresponding authors upon reasonable request.
